# Antimicrobial susceptibility of *Neisseria gonorrhoeae*isolates from symptomatic men attending the Nanjing sexually transmitted diseases clinic (2011–2012): genetic characteristics of isolates with reduced sensitivity to ceftriaxone

**DOI:** 10.1186/s12879-014-0622-0

**Published:** 2014-11-27

**Authors:** Sai Li, Xiao-Hong Su, Wen-Jing Le, Fa-Xing Jiang, Bao-Xi Wang, Peter A Rice

**Affiliations:** STD Clinic, Institute of Dermatology, Chinese Academy of Medical Sciences and Peking Union Medical College, Nanjing, 210042 China; Department of Dermatology, Anhui Provincial Hospital, Hefei, 230001 China; Division of Infectious Diseases and Immunology, University of Massachusetts Medical School, Worcester, 01602 MA USA

**Keywords:** Neisseria gonorrhoeae, Antimicrobial resistance, Resistance plasmids, Ceftriaxone, Resistance determinants

## Abstract

**Background:**

Evolving gonococcal antimicrobial resistance (AMR) poses a serious threat to public health. The aim of this study was to: update antimicrobial susceptibility data of *Neisseria gonorrhoeae* recently isolated in Nanjing, China and identify specific deteminants of antimicrobial resistance and gentoypes of isolates with decreased sensitivity to ceftriaxone.

**Methods:**

334 *N. gonorrhoeae* isolates were collected consecutively from symptomatic men attending the Nanjing STD Clinic between April 2011 and December 2012. The minimum inhibitory concentrations (MICs) for penicillin, tetracycline, ciprofloxacin, spectinomycin and ceftriaxone were determined by agar plate dilution for each isolate. Penicillinase-producing *N. gonorrhoeae* (PPNG) and tetracycline-resistant *N. gonorrhoeae* (TRNG) were examined and typed for β-lactamase and tetM encoding plasmids respectively. Isolates that displayed elevated MICs to ceftriaxone (MIC ≥0.125 mg/L) were also tested for mutations in penA, mtrR, porB1b, ponA and pilQ genes and characterized by *Neisseria gonorrhoeae* multi-antigen sequence typing (NG-MAST).

**Results:**

98.8% (330/334) of *N. gonorrhoeae* isolates were resistant to ciprofloxacin; 97.9% (327/334) to tetracycline and 67.7% (226/334) to penicillin. All isolates were susceptible to ceftriaxone (MIC ≤0.25 mg/L) and spectinomycin (MIC ≤32 mg/L). Plasmid mediated resistance was exhibited by 175/334 (52%) of isolates: 120/334 (36%) of isolates were PPNG and 104/334 (31%) were TRNG. 90.0% (108/120) of PPNG isolates carried the Asia type β-lactamase encoding plasmid and 96% (100/104) of TRNG isolates carried the Dutch type tetM containing plasmid. Elevated MICs for ceftriaxone were present in 15 (4.5%) isolates; multiple mutations were found in penA, mtrR, porB1b and ponA genes. The 15 isolates were distributed into diverse NG-MAST sequence types; four different non-mosaic penA alleles were identified, including one new type.

**Conclusions:**

*N. gonorrhoeae* isolates in Nanjing generally retained similar antimicrobial resistance patterns to isolates obtained five years ago. Fluctuations in resistance plasmid profiles imply that genetic exchange among gonococcal strains is ongoing and is frequent. Ceftriaxone and spectinomycin remain treatments of choice of gonorrhea in Nanjing, however, decreased susceptibility to ceftriaxone and rising MICs for spectinomycin of *N. gonorrhoeae* isolates underscore the importance of maintaining surveillance for AMR (both phenotypic and genotypic).

**Electronic supplementary material:**

The online version of this article (doi:10.1186/s12879-014-0622-0) contains supplementary material, which is available to authorized users.

## Background

Gonorrhea is the fifth most commonly reported infectious disease in China; nearly 100,000 cases were reported officially nationwide in 2013 [1]. Worldwide, over 106 million new infections are diagnosed annually [2]. Effective treatment is key in preventing complications and controlling transmission of *N. gonorrhoeae*. Unfortunately, this bacterium has developed resistance to antimicrobials (AMR) including sulfonamides, penicillins, tetracyclines and quinolones [3],[4]. In recent years treatment failures have occurred when extended-spectrum cephalosporins (ESCs), the most widely used agents for treatment of gonorrhea, were used, and associated increases in AMR to ESCs have been reported in several countries [5]-[11]. The molecular mechanisms associated with reduced susceptibility or resistance to ESCs include mutations in several gene loci; penA [12]-[16], mtrR [17],[18], penB [17],[19], ponA [6],[20] and pilQ [21],[22] and their multifaceted interactions [6],[23].

Several public health organizations have published their action/response plans to control the spread and impact of ESCs-resistant gonococci [2],[24],[25]. These have stressed the importance of enhancing quality-assured surveillance of gonococcal AMR both locally and internationally, with a focus on ESCs.

In China, the surveillance of *N. gonorrhoeae* AMR is conducted by the National Center for Sexually Transmitted Diseases (STDs) Control, China CDC, which systematically collects *N. gonorrhoeae* AMR data from 10 ~ 16 geographically separate monitoring sentinels and provides external quality assessment. Annual AMR rates are available in brief reports issued by China CDC (1987-current) [26] and are also published by the World Health Organization (WHO), Western Pacific Region Gonococcal Antimicrobial Surveillance Programme (1992–current) [27]-[29].

During 2011–2012, the period of this investigation, ceftriaxone and spectinomycin were the only antimicrobial agents recommended officially in the Chinese guidelines for the treatment of gonorrhea [30], however, a wide variety of antimicrobials including (fluoro) quinolones were prescribed unofficially outside of STD Clinics and purchased over the counter to treat gonorrhea.

The Nanjing STD clinic, operating as a sentinel site in Eastern China, performs AMR tests routinely on 10%–20% of gonococcal isolates recovered in this city. We performed this study in 2011–12, to assess the impact, in Nanjing, of recent changes in ceftriaxone susceptibility of *N. gonorrhoeae* that have been reported [31]-[34] and to characterize genetic determinants and NG-MAST sequence types (STs) of isolates with decreased sensitivity to ceftriaxone.

## Methods

### Subjects and bacterial strains

*N. gonorrhoeae* isolates were isolated from male adults with symptoms/signs of urethritis (confirmed by ≥5 polymorphonuclear neutrophils [PMNs]/oil immersion microscopic field seen in all urethral specimens) in the STD clinic of the National Center for STD Control in Nanjing, China between April 2011 and December 2012. These isolates represented 18.1% of the reported gonorrhea cases in Nanjing during this period. Epidemiological data was acquired by interviews conducted by clinical staff using standardized data recording instruments. Isolates from all but 2 men were from ethnic Han (332/334) and 99.4% (332/334) of men were heterosexual. The mean (± SEM) age was 36.7 ± 3.3 years, the median, 36 (range: 19 to 65), there being no significant difference (p > 0.05) compared with the national average age of gonorrhea cases in men (mean [± SEM], 34.9 ± 3.2). 61.3% of men were married. Approval to enroll human subjects was given by the Institutional Review Board of the Institute of Dermatology, Chinese Academy of Medical Science. The study was introduced and explained to potential subjects who voluntarily gave written and signed consent. Urethral specimens were collected with cotton swabs and immediately streaked onto Thayer-Martin (T-M) selective medium. Inoculated plates were incubated at 36°C in candle jars for 24–48 h. *N. gonorrhoeae* was identified by colonial morphology, Gram’s stain, and oxidase testing, which are sufficient to identify *N. gonorrhoeae* colonies isolated on selective medium, particularly for samples from the urethral tracts of symptomatic men [35],[36]. Isolates were subcultured onto GC agar base (Difco, Detroit, MI) supplemented with 1% IsovitaleX™(Oxoid, USA) and pure cultures swabbed, suspended in tryptone-based soy broth and frozen (−70°C) until used for antimicrobial testing.

### Antimicrobial susceptibility testing

The minimum inhibitory concentrations (MICs; mg/L) of *N. gonorrhoeae* to penicillin, tetracycline, ciprofloxacin, spectinomycin and ceftriaxone were determined by the agar dilution method (Clinical Laboratory Standards Institute [37]), using 2-fold serial dilutions of antibiotics (all purchased from Sigma Aldrich, Saint Louis, MO, USA): penicillin 0.125–8 mg/L, tetracycline 0.25–16 mg/L, ciprofloxacin 0.25–8 mg/L, spectinomycin 4–128 mg/L and ceftriaxone 0.004–0.5 mg/L. WHO reference strains A, G, I and J (kindly provided by Dr. J. W. Tapsall, Prince of Wales Hospital, Sydney, Australia) and a ceftriaxone-resistant strain (kindly provided by Carmen Ardanuy, L’Hospitalet de Llobregat, Barcelona, Spain [38]) were used as controls. In addition, we included, as controls, two strains isolated from our prior study that had diminished susceptibiltiy to ceftriaxone (MIC = 0.125 and 0.25 mg/L) [39]. MIC breakpoints for assigning susceptibility, intermediate and resistance status to *N. gonorrhoeae* isolates were determined according to Clinical and Laboratory Standards Institute (CLSI) Document M07-A9 (M100-S22) [37].

Production of β-lactamase was identified by paper acidometric test (phenotype method) [39],[40]. Resistance patterns were defined as follows [41]:

- PPNG (Penicillinase-producing *N. gonorrhoeae*), β-lactamase positive
- TRNG (Plasmid-mediated tetracycline-resistant *N. gonorrhoeae*), MIC to tetracycline ≥16 mg/L, also confirmed by PCR-based genotyping
- PPNG/TRNG, β-lactamase positive and MIC to tetracycline ≥16 mg/L
- PenR (chromosomally mediated resistance to penicillin), non-PPNG, non-TRNG, MIC to penicillin ≥2 mg/L and MIC to tetracycline <2 mg/L
- TetR (chromosomally mediated resistance to tetracycline), non-PPNG, non-TRNG, MIC to tetracycline ≥2 mg/L and MIC to penicillin <2 mg/L
- CMRNG (chromosomal resistance to penicillin and tetracycline), non-PPNG, non-TRNG, MIC to penicillin ≥2 mg/L and MIC to tetracycline ≥2 mg/L.

### Identification and typing of *tetM*and β -lactamase gene producing plasmids

Bacterial DNA was extracted according to a published method [39].

For *tetM* gene containing plasmids, PCR reactions were performed using a universal forward primer; 5′ ^825^CTCGAACAAGAGGAAAGC^842^ and reverse primers 5′^1602^GCATTCCACTTCCCAAC^1586^ to identify the presence of the “American” type *tetM* plasmid and 5′ ^1267^TGCAGCAGAGGGAGG^1253^ to identify the “Dutch” type. PCR products were separated by agarose electrophoresis, stained with ethidium bromide and visualized using ultraviolet fluorescent light for the presence of a 778 bp fragment (“American”) or a 443 bp fragment (“Dutch”) [42]. An American type *tetM* containing strain identified in our previously study [39] and WHO reference strain G (Dutch type) were used as positive controls.

β-lactamase producing plasmids were characterized using multiplex PCR (employing specific forward and reverse primers [43]) followed by elecrophoresis to identify specifically sized PCR products, thereby differentiating three common plasmids: Asian (958 bp), African (1191 bp) and Toronto (650 bp). Positive control PCR products derived from plasmids pJD4 (Asian type), pJD5 (African type) and pJD7 (Toronto type) (provided by J.R. Dillon, University of Saskatchewan, Canada) were included in each assay.

### Sequencing of *N. gonorrhoeae*genetic determinants associated with decreased susceptibility to ceftriaxone

Amplification of *penA*, *mtrR*, *porB* (and also *ponA* and *pilQ*) was performed using published primers and conditions [44],[45]. PCR products were sequenced twice bidirectionally on an Applied Biosystems 3730XL DNA automatic sequencer. Nucleotide and deduced amino acid sequences were edited and aligned against their respective prototypes [44]. A newly identified PBP2 type was designated XLI according to previously used nomenclature [12],[15],[46]-[49].

### Molecular epidemiological typing

*N. gonorrhoeae* multiantigen sequence typing (NG-MAST) was performed by PCR amplification and sequencing of two high polymorphic fragments in *porB* and *tbpB* genes according to previously described methods [50]. Allele numbers and sequence type numbers were assigned through the NG-MAST database (http://www.ng-mast.net).

### Nucleotide sequence accession numbers

*porB* sequences were submitted to the GenBank nucleotide database under accession numbers [Genbank: KF660589-KF660594] and [Genbank: KF668353- KF668360]. Novel *penA* nucleotide sequences associated with reduced susceptibility to ESCs identified in this study were deposited in the GenBank database under accession number [Genbank: KF576657].

### Statistical analysis

The Chi-Square test (2-tailed) was used to assess significance of associations and compare resistance rates; p values <0.05 were considered significant. Statistical analyses were performed using IBM SPSS Statistics version 22.0.

## Results

### Antimicrobial susceptibilities

Among 652 consecutively enrolled male subjects with urethritis, 340 (52.1%) were infected with *N. gonorrhoeae*; antimicrobial susceptibility testing of 334 successfully retrieved isolatesis is summarized in Table 1 (six isolates were not recovered from storage or could not be separated from contaminants). Resistance rates to antibiotics formerly or now recommended for treatment of gonorrhea in China were: ciprofloxacin 98.8%, tetracycline 97.9%, penicillin 67.7%, all isolates were susceptible to spectinomycin and ceftriaxone. The minimum inhibitory concentration of 50% and 90% of gonococcal isolates (MIC_50_/MIC_90_) for ciprofloxacin, tetracycline, penicillin, spectinomycin and ceftriaxone were >8/>8, 8/>16, 2/>8, 16/32 and 0.03/0.06 mg/L, respectively.

**Table 1:** ***In vitro*** **antimicrobial susceptibility of** ***Neisseria gonorrhoeae*** **: clinical isolates (n = 334)**

Antimicrobial breakpoints (susceptible/resistant [mg/L]) ^a^	No. (%)			MIC (mg/L)		
	Susceptible	Intermediate	Resistant	Range	MIC _50_	MIC _90_
Penicillin G (S ≤ 0.06/R ≥ 2)	0	108 (32.3)	226 (67.7)	0.125 to >8	2	>8
Tetracycline (S ≤ 0.25/R ≥ 2)	2 (0.6)	5 (1.5)	327 (97.9)	0.25 to >16	8	>16
Ciprofloxacin (S ≤ 0.06/R ≥ 1)	0	4 (1.2)	330 (98.8)	≤0.25 to >8	>8	>8
Spectinomycin (S ≤ 32/R ≥ 128)	334 (100)	0	0	≤4 to 32	16	32
Ceftriaxone (S ≤ 0.25)	334 (100)	-	-	0.004 to 0.25	0.03	0.06

^a^Antimicrobial breakpoints that distinguish susceptible from resistant gonococcal isolates (CLSI Document M07-A9 (M100-S22) [35]. MIC: minimum inhibitory concentration.

### Penicillin and tetracycline resistance phenotypes

Penicillin and tetracycline resistance phenotypes were categorized based on plasmid or chromosomally mediated resistance mechanisms. Overall, plasmid mediated resistance to either penicillin or tetracycline was exhibited by 175/334 (52%): 71/334 (21%) of isolates were PPNG exclusively; 55/334 (16%) were TRNG exclusively and 49 (15%) were PPNG/TRNG. CMRNG or TetR alone was identified in 27.8% and 18.3% of isolates, respectively. PenR alone was not detected.

### Molecular typing of PPNG and TRNG

120 β-lactamase producing *N. gonorrhoeae* isolates were distributed into two plasmid types: Asian and African. No Toronto plasmid was detected. 108 (90%) isolates contained the Asian plasmid and 12 (10%) harbored the African plasmid.

Among 104 isolates with the *tetM* determinant, 96.2% (100/104) carried the Dutch type; the remaining 4 carried the American-type.

The β-lactamase plasmid was present more frequently in TRNG isolates than in non-TRNG isolates (47.1% [49/104] vs. 30.9% [71/230]; p < 0.01; Table 2). Forty nine PPNG/TRNG isolates exhibited two patterns that combined β-lactamase and *tetM* containing plasmids: Asian type β-lactamase expressing isolates always harbored the Dutch *tetM* type; the combination of African type β-lactamase and American type *tetM* was rarely identified in PPNG/TRNG (3 isolates) in Nanjing (Table 2).

**Table 2:** **Distribution of β-lactamase-encoding and** ***tetM*** **-encoding plasmids:** ***Neisseria gonorrhoeae*** **clinical isolates (n = 334)**

Type of ***tetM*** -encoding plasmid	Type of β-lactamase producing plasmid		
	Asian/New Zealand	African/Nîmes	β-lactamase negative
Dutch	46	0	54
American	0	3	1
*tetM* negative	62	9	159

### Genetic characteristics of *N. gonorrhoeae*isolates with increased MIC to ceftriaxone

Fifteen (4.5%) isolates had elevated MICs (≥0.125 mg/L) to ceftriaxone. A second determination on the 15 isolates confirmed the MICs. According to recent CLSI breakpoint criteria [37], these isolates would be classified as “susceptible” but treatment failures have occurred when infected strains possessed MICs around 0.125 mg/L [6],[10],[11]. We examined these 15 isolates for genetic mutations in *penA*, *mtrR*, *penB*, *ponA* and *pilQ* genes (Table 3).

**Table 3:** **Genetic characteristics of** ***Neisseria gonorrhoeae*** **with increased MICs (n = 15) to ceftriaxone (≥0.125 mg/L)**

Isolate no.	Ceftriaxone MICs (mg/L)	Antimicrobial characteristics	NG-MAST			PBP2 allele	Polymorphisms in:			
			porB	tbpB	STs		MtrR	PorB1b	PBP1	PilQ
NJ-1	0.25	CMRNG, CipR^#^	5658	111	9530*	XVIII	−57A del, A39T	G120K, A121D	L421P	wt
NJ-2	0.25	CMRNG, CipR	5175	328	8737	XVIII	−57A del, A39T	G120K, A121D	L421P	wt
NJ-3	0.125	PPNG, CipR	5659	107	9531*	XVIII	−57A del	G120K, A121G	L421P	wt
NJ-4	0.125	PP/TRNG, CipR	1053	186	3289	XVIII	−57A del	G120K, A121D	L421P	wt
NJ-5	0.125	PPNG, CipR	1854	33	9532*	XLI**	−57A del	G120N, A121D	L421P	wt
NJ-6	0.125	PP/TRNG, CipR	543	294	9533*	XVIII	A39T	G120K, A121G	L421P	wt
NJ-7	0.125	CMRNG, CipR	5660	186	9534*	XVIII	−57A del	G120K, A121G	L421P	wt
NJ-8	0.125	CMRNG, CipR	505	135	2186	XVIII	−57A del	G120K, A121D	L421P	wt
NJ-9	0.125	CMRNG, CipR	1285	4	9535*	XIII	−57A del, G45D	G120K, A121D	L421P	wt
NJ-10	0.125	TRNG, CipR	4	831	3746	XIII	−57A del	G120K, A121D	L421P	wt
NJ-11	0.125	CMRNG, CipR	3530	4	9536*	XVIII	−57A del	G120K, A121G	L421P	wt
NJ-12	0.125	CMRNG, CipR	1198	156	2461	XVIII	−57A del, A39T	G120K, A121G	L421P	wt
NJ-13	0.125	CMRNG, CipR	5661	4	5061	XVIII	−57A del, G45D	G120K, A121N	L421P	wt
NJ-14	0.125	CMRNG, CipR	2978	1058	9537*	XII	−57A del	G120K, A121D	L421P	wt
NJ-15	0.125	TRNG, CipR	543	438	9538*	XVIII	−57A del	G120K, A121G	L421P	wt

wt, wild type.

*New NG-MAST STs detected in this study.

**A new PBP2 allele previously not identified was named XLI according to previously used Nomenclature.

^#^isolates exhibiting resistance to ciprofloxacin (MIC ≥ 1 mg/L).

Four PBP2 amino acid sequence patterns were identified; PBP2 allele XVIII was the predominant type (n = 11), followed by XIII (n = 2) and XII (n = 1). A new pattern containing five mutations (345D insertion, A501V, F504L, A510V and A516G) was designated PBP2 allele XLI, according to the previously used nomenclature of PBP2 alleles in *N. gonorrhoeae* [12],[15],[46]-[49]; none of these is a mosaic allele. However, 12 isolates (allele XVIII and XLI) possessed an A501T substitution in PBP2, and 2 isolates (allele XIII) had an A501V substitution.

Fourteen of fifteen isolates contained a single nucleotide (A) deletion in the 13 bp inverted repeat motif located between −10 and −35 sequence in the *mtrR* promoter. Five isolates (including two with ceftriaxone MICs = 0.25 mg/L) also had a G45D or A39T substitution in the DNA-binding motif of MtrR, and one isolate possessed a single amino acid replacement (A39T). We did not identify the recently described C-to-T mutation 120 bp upstream of the *mtrC* start codon, termed *mtr*_*120*_ [51].

All isolates possessed *penB* resistance determinants with substitutions at both G120 and A121 in loop 3 of the PorB1b outer membrane porin; these included G120K/A121D (n = 7), G120K/A121G (n = 6), G120N/A121D (n = 1) and G120K/A121N (n = 1).

Finally, the L421P substitution in PBP1 caused by a single nucleotide mutation (T to C) in the *ponA* gene was detected in all isolates; we saw no alternations in the *pilQ* gene.

The fifteen isolates that harbored 14 different *porB* sequences and 12 *tbpB* sequences were assigned to 15 different NG-MAST STs: ST8737, ST3289, ST2186, ST3746, ST2461, ST5061 and 9 new STs. Eleven isolates with the PBP2 allele, XVIII, the most common PBP2, were resolved into 11 separate NG-MAST STs (Table 3).

## Discussion

Our study reported high-levels of resistance to previously recommended therapeutic agents for *N. gonorrhoeae* of isolates from men with urethritis in Nanjing (2011–12): ciprofloxacin (98.8%); tetracycline (97.9%) and penicillin (67.7%). Rates of resistance have remained unchanged compared with that of 2006, the last year of a six yearly sequential measurement [39]. Similar patterns of resistance have also been noted from other cities in southern China; Shanghai (ciprofloxacin 98.7%, tetracycline 73.6%, penicillin 98.1% [exact time period for isolates collection not available]) [52], Shenzhen (ciprofloxacin 96.1%, tetracycline 99.0% in 2008–2012) [53], Hainan (ciprofloxacin 98.0% in 2011) [54] and other western pacific and southeastern Asian regions including Vietnam, Korea, India, Pakistan and Bhutan [28],[31],[55]-[57]. The contribution of plasmid mediated resistance was similar in Nanjing in the period 2011–12 compared to 2006 [39], but during this interval declines in the proportion of PPNG have been observed in Korea [57], Canada [58], Latin America and the Caribbean countries [59]. Because treatment of gonorrhea with ciprofloxacin, tetracycline and penicillin has long been discontinued in Nanjing [30],[39], persistence of resistant phenotypes may be explained by the stability of mechanisms that promote resistance or the presence of unidentified selection pressures due to use of antibiotics for other reasons including the common practice of self-medication with antibiotics. In our study, 33% of men with gonococcal urethritis reported self-administration of antibiotics in the 30 days prior to their clinic visit (unpublished data).

**Figure 1:**
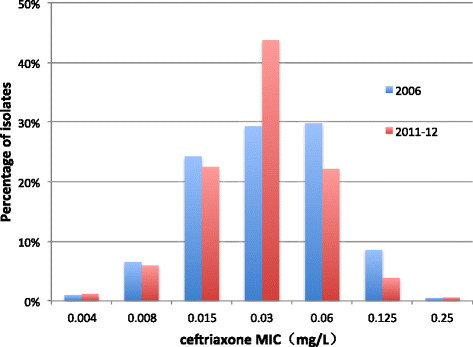
**Distribution of ceftriaxone MICs of** ***Neisseria gonorrhoeae*** **isolates: Nanjing, 2006 (n = 198) and 2011–12 (n = 334).**

**Figure 2:**
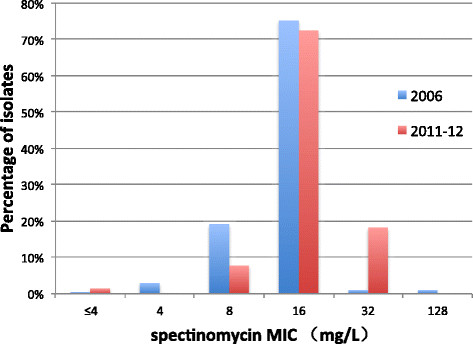
**Distribution of spectinomycin MICs of** ***Neisseria gonorrhoeae*** **isolates: Nanjing, 2006 (n = 198) and 2011–12 (n = 334).**

It is encouraging to note that the percentage of gonococci isolates with decreased susceptibility to ceftriaxone (MIC ≥0.125 mg/L) declined significantly from 9.1% (18/198) in 2006 [39] to 4.5% (15/334) in 2011–12 (p = 0.04); a one dilution decrease of the modal MIC for ceftriaxone was also observed (0.06 mg/L in 2006 to 0.03 mg/L in 2011–12) (Figure 1). In Europe [32],[60]-[63], Australia [64], Africa [65]-[67], the western hemisphere [33],[58],[59],[68]-[70] and the South-East Asia Region [31], MICs to ceftriaxone have risen during this period. Nonetheless, it is of concern that the distribution of spectinomycin MICs shifted upward in Nanjing during the same interval, between 2006 and 2011–12 (Figure 2) [39]. In 2006, an MIC = 32 mg/L to spectinomycin was observed in 1.0% of strains, rising to 18.3% in 2011–12 (p < 0.01), coinciding with a significant decrease in the percentage of strains whose MICs ≤8 mg/L (p < 0.01). Opposing trends in resistance of gonococcal isolates to ceftriaxone and spectinomycin may, in part, result from the greater use of spectinomycin in the Nanjing STD clinic. Ninety-eight percent of gonococcal urethritis cases in men were prescribed spectinomycin as recently as in 2012, in large part because of the lower price of Chinese-manufactured spectinomycin vs. imported ceftriaxone and the requirements for cefriaxone skin testing prior to administration. Resistance of *N. gonorrhoeae* to spectinomycin, although rarely reported [31],[33],[65],[71], because of limited access to and use of this antibiotic in many countries outside of China, may be the “cautionary tale” of clinical failure when it is used as primary treatment of gonorrhea [72].

Plasmids carried by PPNG and TRNG isolates have changed over time in Nanjing [39]. The highly active TEM β-lactamase gene is carried by a family of plasmids in *N. gonorrhoeae*. Since 1976, at least eight different plasmids have been identified from geographically distinct locations [73]. The β-lactamase gene has been found on Asian-, African- and Toronto - type plasmids carried by epidemic strains of *N. gonorrhoeae* [74], even while new types such as the Johannesburg and Australian plasmids are being discovered [75],[76]. We did not differentiate the Nîmes plasmid from the African-type or the New Zealand plasmid from the Asian-type, owing to the identical sizes of the PCR products but we would have identified the novel Johannesburg plasmid identified by the 458 bp PCR products using the primer pair designed for the African-type plasmid. Using a simple multiplex PCR method [43], we detected either Asian/ New Zealand type (n = 108) or African/Nîmes type (n = 12) plasmids in all penicillinase-positive isolates. The Asian/New Zealand type plasmid dominated, while the newly emerging African/Nimes type, which was absent in Nanjing isolates of 2006, and were epidemic in the United Kingdom (UK) and West Africa [74], may have reflected movement of *N. gonorrhoeae* across continents. In like manner, while the Dutch variant (n = 100) of *tetM* still predominates, identification of the minor American variant (n = 4) in Nanjing is just beginning.

Decreased susceptibility to ceftriaxone was demonstrated in 15 (4.5%) of the Nanjing isolates of *N. gonorrhoeae* (cefixime was not tested/not used in Nanjing). Differing from gonococcal isolates that were resistant or less susceptible to ESCs from Japan [77], Europe [10],[38],[78]-[80], North America [47],[81] or South Africa [67], the mosaic-like structure that is usually located in the region of the transpeptidase-encoding domain of *penA* of resistant gonococcal strains, was not identified in the Nanjing isolates. Similar to strains recently tested from Southeast Asia (Vietnam) [56] and South Asia [55], the majority of our 15 isolates with decreased susceptibility contained mutations in PBP2 at position 501: A501V or A501T. Whiley et al. [13] have suggested that gonococcal-specific A501 substitutions may contribute more significantly to reduced ceftriaxone susceptibility than mosaic sequences. Three A501 mutation-containing patterns of PBP2 (XVIII, XIII and XLI) in our study have been reported to be epidemiologically connected with increased MICs to ceftriaxone [15],[44],[45]. The results of a modeling study indicated that reduced susceptibility to cephems such as cefixime and ceftriaxone is due to a conformational alteration of the beta-lactam-binding pocket caused by an A501 mutation in non-mosaic strains [13]. Results of the model have been confirmed by isogenic transformation demonstrating, that an A501V(T) mutation in PBP2 results in increased MIC to ceftriaxone in a non-mosaic penA containing strain [82]. Polymorphisms in *mtrR* and *penB* genes (and also *ponA*) were also evident in our isolates.

Azithromycin, combined with ceftriaxone, recently has been recommended by the United States [83] and the United Kingdom [84] and is now also in the European guidelines [85] for the treatment uncomplicated gonorrhea. This combination had not been recommended for the treatment of gonorrhea in China during the study period (2011–2012). Azithromycin resistance of *N. gonorhoeae* isolates from the Nanjing STD clinic was 6.8% [86] in 2008 and 2009, which exceeded the 5% threshold suggested by WHO for emperic use as a first line agent [26]-[29].

Our study has several limitations. Gonococcal isolates were collected only from men with symptomatic urethritis. 33% of infected men had used antibiotics prior to their clinic visit, which may have contributed to increased resistance of their gonococcal isolates. Exclusion of isolates from asymptomatic men and from extragential sites (there were only two MSMs in our study) may have underestimated AMR [87]-[89]. Simultaneously, infected men may have been cured of infection with isolates sensitive to the antibiotics that they had used earlier and therefore not seen in the clinic. This may have also led to an overabundance of more resistant *N. gonorrhoeae*.

## Conclusions

This study confirmed that ceftriaxone and spectinomycin remain effective empiric first-line therapy for gonorrhea in Nanjing. However, years of discontinued use of formerly recommended antimicrobial agents did not restore the sensitivities of gonococcal strains to ciprofloxacin, penicillin or tetracycline. Changes and multiple representative types of resistance plasmid profiles highlight frequent influx and exchange of *N. gonorrhoeae* strains. Reduced ceftriaxone susceptibility was found in 4.5% of gonococcal isolates and alterations in *penA*, *mtrR*, and *penB* genes were important determinants of resistance. Sustained surveillance of gonococcal phenotypic and genotypic AMR is an important element in the control of gonoccocal infection.

## Authors’ original submitted files for images

Below are the links to the authors’ original submitted files for images. Authors’ original file for figure 1 Authors’ original file for figure 2

## Abbreviations

AMR: Antimicrobial resistance
PPNG: Penicillinase-producing *N. gonorrhoeae*
TRNG: Tetracycline-resistant *N. gonorrhoeae*
MIC: Minimum inhibitory concentration
NG-MAST: *Neisseria gonorrhoeae* multi-antigen sequence typing
ST: Sequence type
STI: Sexually transmitted infection
STD: Sexually transmitted diseases
ESC: Extended-spectrum cephalosporin
WHO: World Health Organization
CLSI: Clinical and Laboratory Standards Institute
PBP: Penicillin-binding protein
