# Supplementary figures and images for: Antimicrobial susceptibility of Neisseria gonorrhoeaeisolates from symptomatic men attending the Nanjing sexually transmitted diseases clinic (2011–2012): genetic characteristics of isolates with reduced sensitivity to ceftriaxone
Source: BMC Infect Dis. 2014 Nov 27;14:622. doi: 10.1186/s12879-014-0622-0 (PMC4263019; doi:10.1186/s12879-014-0622-0)

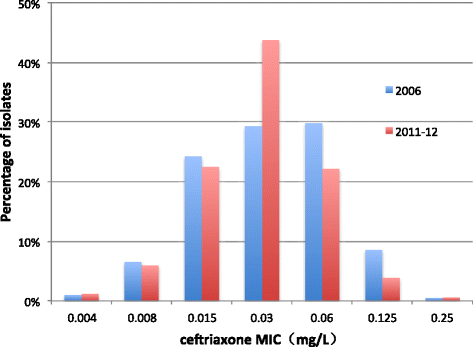

Supplement: Supplementary file 1 — Authors’ original file for figure 1 [file 12879_2014_622_MOESM1_ESM.gif]

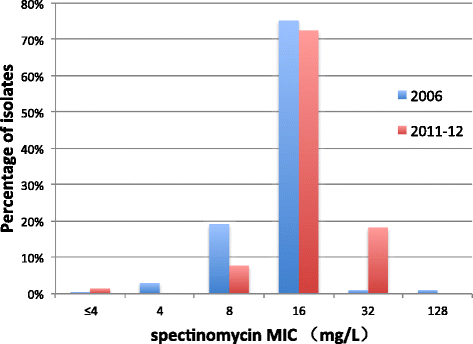

Supplement: Supplementary file 2 — Authors’ original file for figure 2 [file 12879_2014_622_MOESM2_ESM.gif]
